# Mode of Action of Antimicrobial Potential Protease SH21 Derived from *Bacillus siamensis*

**DOI:** 10.3390/ijms25137046

**Published:** 2024-06-27

**Authors:** Hasan Tarek, Seung Sik Cho, Kyung Bin Nam, Ji Min Lee, Sang Hun Lee, Jin Cheol Yoo

**Affiliations:** 1 Department of Pharmacy, College of Pharmacy, Chosun University, Gwangju 61452, Republic of Korea; hasantarek93@gmail.com (H.T.); nchw810@naver.com (K.B.N.); jim1235123@naver.com (J.M.L.); snickers0486@naver.com (S.H.L.); 2 Department of Pharmacy, College of Pharmacy, Mokpo National University, Muan 58554, Republic of Korea; sscho@mokpo.ac.kr; 3 Department of Biomedicine, Health & Life Convergence Sciences, BK21 Four, Biomedical and Healthcare Research Institute, Mokpo National University, Muan 58554, Republic of Korea

**Keywords:** protease SH21, antimicrobial agents, microbial infections, PI uptake, membrane permeability

## Abstract

Global public health is facing a major issue with emerging resistance to antimicrobial agents. Antimicrobial agents that are currently on the market are strong and efficient, but it has not been ruled out that these medications will eventually cause resistance to bacteria. Exploring novel bioactive compounds derived from natural sources is therefore, crucial to meet future demands. The present study evaluated the mode of action of the antimicrobial potential protease enzyme SH21. Protease SH21 exhibited antimicrobial activity, strong heat stability (up to 100 °C), and pH stability (pH 3.0 to 9.0). In terms of mode of action, we found that protease SH21 was able to disrupt the bacterial cell membrane as the results of the nucleotide leakage and cell membrane permeability assay. In addition, we also checked inner membrane permeability by PI uptake assay which suggested that protease SH21 has the ability to enter the bacterial cell membrane. Our results revealed that the antimicrobial protease SH21 might be a promising candidate for treating microbial infections.

## 1. Introduction

In recent years, bacterial infections have emerged as a major public health concern. One of the most significant issues in the world now is antibiotic resistance, which has spread like a pandemic. Antimicrobial resistance (AMR) is caused by alterations in the features of bacteria and thus antibiotic may lose or reduce their efficacy. Antibiotic-resistant bacteria will grow and spread until they become more harmful since antibiotics cannot kill them. The resistance of viruses, bacteria, parasitic organisms, and other infectious agents currently seriously threatens the control of infectious diseases. In recent times, there has been a rise in the prevalence of bacterially induced infectious diseases in correlation with expanding global populations [1]. Some specific Gram-positive and Gram-negative bacteria can cause human infections. Despite the effectiveness of current antibacterial agents, the emergence of resistance to them is not ruled out. Thus, it is necessary to discover new antibacterial drugs [2]. In the past ten years, there has been a significant amount of research conducted concerning novel bioactive chemicals identified in nature. A common Gram-positive bacterium of the skin and human respiratory system is *Staphylococcus aureus*, which is a cause of skin infections, respiratory illnesses, and food poisoning even though it is not necessarily harmful. Resistant strains of bacteria have emerged and spread as a result of the widespread use of antibiotics to treat bacterial illnesses. *S. aureus* has effectively developed a variety of resistance mechanisms to withstand the effects of almost all antibiotics [3].

Since methicillin resistance causes community-acquired methicillin-resistant *S. aureus* (CA-MRSA) is now frequently reported, the emergence of new medications or alternative therapies must be developed. The potential for developing biologically active proteins or peptides as antibacterial agents has drawn the attention of researchers. These compounds are thought to be generally classified into primary metabolites. Koreans have been eating fermented and salted vegetables like kimchi for over 2000 years. Kimchi is a rich source of vitamins, minerals, dietary fiber, and other beneficial components. Research has indicated that kimchi possesses antibacterial, antioxidant, anticancer, antidiabetic, and anti-obesity properties. Proteolytic enzymes, which are essential for effectively hydrolyzing proteins to produce bioactive peptides, are mostly obtained from microbial fermentation. Proteases are diverse families of hydrolytic enzymes that control both infectious illnesses and metabolism [4]. In comparison to other extracellular factors, some researchers have even classified them as the primary virulence factors [5]. The bacterial protease *Xylariapsidii* KT30 is antagonistic against both *S. aureus* and *Bacillus subtilis*, and it has antibacterial potential [6]. Protease from marine *Bacillus subtilis* has also been shown to have antibiotic activity against *Arthrobacter* sp., *Micrococcus luteus*, *S. aureus*, *Bacillus pumilus*, and *Klebsiella pneumoniae* [7]. In our previous studies, we already described various biochemical characterizations, antimicrobial, anti-inflammatory, antioxidant, and anticancer properties of protease SH21, which revealed the unique characteristics of our purified enzyme [8,9]. The purpose of this work was to elucidate the mechanism of action of protease SH21, an antibacterial prospective candidate for use in medical fields, especially in microbial infections.

## 2. Result and Discussion

### 2.1. Growth and Antimicrobial Activity Curves of Protease SH21

**Figure 1:**
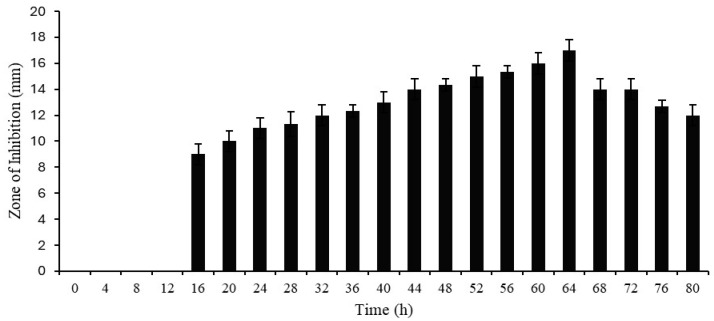
Growth and antimicrobial activities of Protease SH21 (*E. coli* used as indicator bacterial strain).

The antimicrobial activity of cell-free supernatant was detected at 16 h with a zone of inhibition of 9 mm. The antimicrobial activity gradually increased and showed the highest activity at 64 h, with a zone of inhibition of around 17 mm. After that, activity was steadily reduced up to 80 h (Figure 1). 

### 2.2. Effect of pH and Temperature on the Antibacterial Activity of Protease SH21

The effect of pH and temperature on the antimicrobial activity of protease SH21 is shown in Table 1. Subjecting Protease SH21 to pH 3.0, 6.0, and 9.0 for 2 h showed no effect on the antimicrobial activity of SH21. However, its antimicrobial activity was completely lost after 2 h at pH 12.0. Protease SH21 was found to be heat stable at 30, 40, and 50 °C, with a 16 mm zone of inhibition, and at 60,70, 80, and 100 °C, the antimicrobial activity was slightly reduced to 15, 14, 13, and 12 mm, respectively. But, after heating at 121 °C, protease SH21 lost all activity. According to these findings, protease SH21 showed good heat stability, which may help increase its range of applications.

### 2.3. Cell Membrane Permeability of Bacteria by Protease SH21

**Figure 2:**
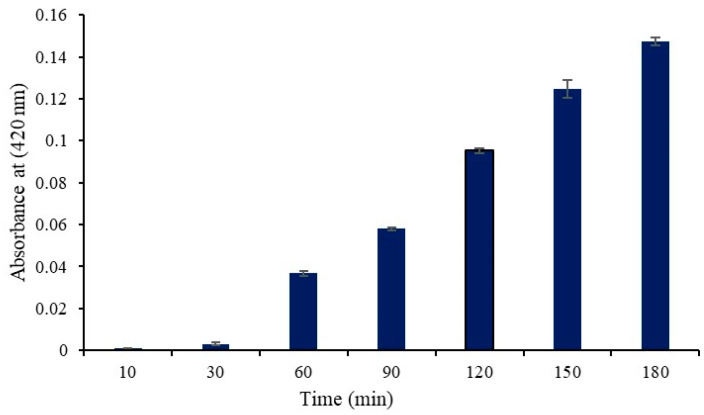
β-Galactosidase activity (Abs at 420 nm) in the culture medium of *S. aureus* cells treated with protease SH21. Data stated as mean ± standard deviation (*N* = 3).

According to a prior study, *S. aureus* cells may generate β-galactosidase in their cytoplasm when lactose and galactose are present in the growth media [10]. Extracellular cytoplasmic β-galactosidase can be identified if the cell membrane is compromised because the enzyme permeates through the damaged membrane. In the current work, we induced the production of cytoplasmic β-galactosidase in *S. aureus* using M9 lactose medium. Protease SH21 was added to the culture medium, and then, the enzymatic activity of b-galactosidase was evaluated. As illustrated in Figure 2, β-galactosidase activity was observed in the culture medium 30 min following protease SH21 treatment, and it continued to increase for the duration of the test (up to 180 min). Protease-SH21-treated control bacterial cells did not exhibit any β-galactosidase activity in their growth medium. These findings suggested that protease SH21 enhanced *S. aureus* cell membrane permeability, causing cytoplasmic content to enter the culture medium.

### 2.4. Nucleotide Leakage of Bacteria by Protease SH21

**Figure 3:**
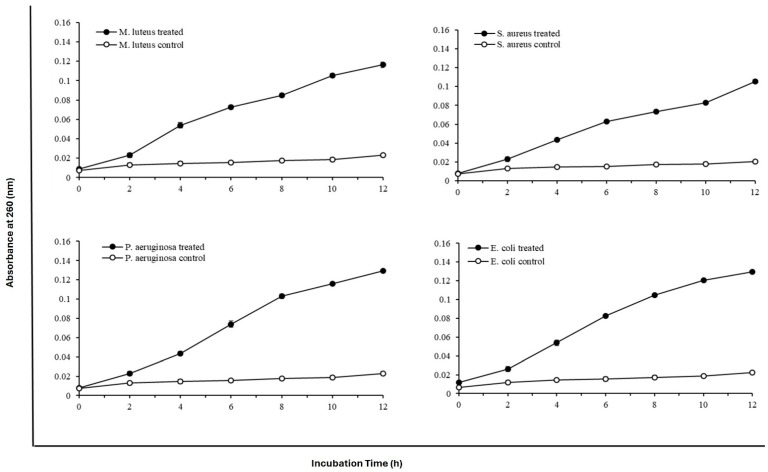
Effect of protease SH21 on nucleotide leakage of different Gram-positive and Gram-negative bacteria at different time intervals. Data stated as mean ± standard deviation (*N* = 3).

The effects of protease SH21 on the total amount of nucleotide leakages of four different bacterial cells at different intervals were studied and are shown in Figure 3. The activity of protease SH21 on bacterial membrane permeability was determined against strains by calculating OD at 260 nm at different intervals. Protease SH21 showed almost the same nucleotide leakage activity against all tested bacteria. *M. luteus* and *S. aureus* showed a steady increase in OD value, which reached a maximum of 0.12 and 0.11, respectively, at 12 h. All bacterial strains treated with the purified SH21 showed an increase in the value at OD 260 nm within 12 h. Thereafter, the protease SH21 showed maximum absorbance of 0.129 and 0.13 at OD 260 nm at 12 h in the case of *P. aeruginosa* and *E. coli*, respectively. Overall, our results are consistent with the antimicrobial protease research published by Muthu et al. [11].

### 2.5. Membrane Integrity of Bacteria by Protease SH21

**Figure 4:**
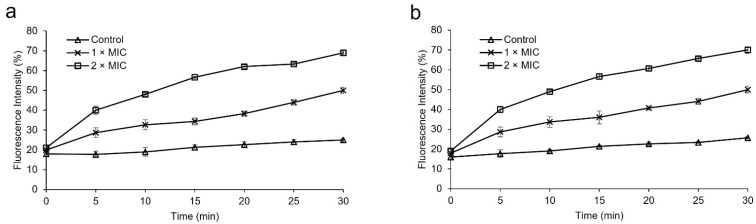
PI uptake assay for membrane integrity of bacteria by protease SH21 (**a**) *E. coli* ATCC 1923, (**b**) *S. aureus* KCTC 1928. All experiments were carried out three times.

SEM, TEM, and PI Uptake assay are commonly used to evaluate the degree of bacterial membrane damage. In this work, we used the fluorescent dye PI to investigate the effects of protease SH21 on the inner membrane of bacterial strains (*E. coli* and *S. aureus*). The peptide treatment usually made it possible for PI to enter bacterial cells. The fluorescence intensity increased over time when protease at 1× MIC and 2× MIC was present, as demonstrated in Figure 4a,b. The protease SH21 showed the maximum fluorescence against *E. coli* and *S. aureus* at 2× MIC, of roughly 69% and 70%, respectively. The outcome suggested that the integrity of the bacterial cell membrane was impacted by the protease SH21.

## 3. Materials and Methods

### 3.1. Materials

Protease SH21 was isolated from Korean fermented food kimchi. o-nitrophenyl-β-D-galactopyranoside (ONPG) was purchased from Sigma-Aldrich (St. Louis, MO, USA). Analytical-grade reagents were used in all experiments.

### 3.2. Screening, Identification, and Isolation of Bacterial Strain

Isolation of protease-producing strain *Bacillus siamensis* CSB55 was described in our previously published paper by Tarek et al. [8].

### 3.3. Production and Purification of Protease SH21

Production and purification of protease SH21 were carried out according to our previous reports [8,9]. *Bacillus siamensis* CSB55 was used in the medium (g/L) for protease production: Casein 10, peptone 5, tryptone 5, MgSO_4_·7H_2_O 0.1, KH_2_HPO_4_ 0.2, CaCl_2_ 0.1, and KH_2_HPO_4_ 0.1; pH 8.5. Seed culture inoculum was prepared using Luria–Bertani (LB) medium containing (g/L) 10 peptone, 5 yeast extract, and 0.5 NaCl, pH 7.0, autoclaved at 121 °C for 20 min. The 1% seed culture was added to a 2 L Erlenmeyer baffle flask with 300 mL of production medium and incubated at 37 °C for 64 h with shaking at 160 rpm. The incubated medium was centrifuged at 10,000 rpm for 30 min at 4 °C to obtain crude protease. The cell-free supernatant was purified with ammonium sulfate (40–80%), centrifuged again, and resuspended in Tris-HCl buffer (20 mM, pH 9.0). The enzyme sample was applied to a Sepharose CL-6B column (80 cm × 1.8 cm) and equilibrated with Tris-HCl buffer (20 mM, pH 9.0). The concentrated sample was loaded into a Sephadex G-75 column (20 cm × 2.0 cm) and eluted with the same buffer. The active protease fractions were collected and then lyophilized.

### 3.4. Protease Assay

Protease activity was determined according to the previously described method [12]. Briefly, 350 μL enzyme solution (20 ug/mL) was mixed with the substrate (0.5% azocasein in 20 mM Tris-HCl buffer, pH 9.0). The enzyme–substrate solution was then incubated at 55 °C for 30 min. After that, 500 μL of 10% (*v*/*v*) TCA solution was added to stop the reaction. To remove undigested protein, the solution was centrifuged at 10,000 rpm for 10 min. Then, 200 μL of supernatant solution was combined with 800 μL 1 N NaOH, and finally, absorbance was taken at 440 nm. The amount of enzyme activity that causes a change in the optical density of 0.01 at 440 nm under standard assay conditions was defined as one unit (U) of protease activity.

### 3.5. Determination of Growth and Antimicrobial Activity Curves of Protease SH21

Protease production media was used to produce the protease enzyme from *Bacillus siamensis* CSB55, and casein was added to the medium as a protease inducer. A 250 μL Erlenmeyer baffle flask carrying 50 mL of protease production media was added with a 1% seed culture. It was then incubated for 80 h at 37 °C while being constantly shaken at 160 rpm and cultured broth was taken out every 4 h. The cultured medium was centrifuged for 30 min at 10,000 rpm resulting in cell-free supernatants, which were then utilized to measure the antimicrobial activity in terms of zone of inhibition (mm) every 4 h for 80 h using *Escherichia coli* as the indicator strain.

### 3.6. Effect of pH and Temperature on the Antimicrobial Activity of Protease SH21

Protease SH21 was incubated at various pH and temperature levels to examine the impact of these factors on its antibacterial activity. In an aqueous solution, the optimum pH for protease SH21 was around 9.0 [8]. The pH sensitivity tests of the protease SH21 were performed at pH values of 3.0, 6.0, 9.0, and 12.0 at 37 °C for 2 h. The samples were readjusted to the optimum pH of 9.0 following the treatment in several pH tests to determine the impact of changing the pH on antibacterial activity. For the heat treatment tests, the enzyme was incubated at 30, 40, 50, 60, 70, 80, 100, and 121 °C for 60 min. The antimicrobial activity of all the treated samples was determined by the zone of inhibition (mm) with *E. coli* as the indicator strain. 

### 3.7. Cell Membrane Permeability Assay

To determine the permeability of the cell membrane, the cytoplasmic β-galactosidase released from *S. aureus* cells into the culture medium was measured using ONPG as the substrate, following previously described methods with minor modifications [10,13,14,15]. *S. aureus* cultures were incubated in M9 lactose medium [16] after being centrifuged at 3000× *g* following an overnight incubation at 37 °C in Luria–Bertani broth. The bacterial cells were collected and centrifuged at 3000× *g* for 1 min. They were then washed twice with sterile saline and resuspended in the assay buffer (0.8 g NaCl, 0.02 g KCl, 0.29 g Na_2_HPO_4_, 0.024 g KH_2_PO_4_, 0.025 g MgSO_4_, and 0.39 g β-mercaptoethanol dissolved in 100 mL of double-distilled water). Finally, ONPG was added to the mixture up to the final concentration was 0.1 mg/L. Protease SH21 was then added, with the cell suspension without Protease SH21 serving as the control, reaching a final concentration of 1× MIC. At 37 °C, the cell suspension was cultured. The o-nitrophenol production was observed using a microplate reader set at 420 nm over a period of time.

### 3.8. Nucleotide Leakage of Bacteria by Protease SH21

The experiment was carried out, with some modifications, according to Xiao et al. [17]. The antibacterial activity of SH21 against several Gram-positive and Gram-negative bacteria [8] was examined in our earlier study in terms of the minimum inhibitory concentration (MIC). To investigate the nucleotide leakage in the bacteria, the four bacterial strains—*Micrococcus luteus* ATCC 9341, *Staphylococcus aureus* KCTC 1928, *Pseudomonas aeruginosa* KCTC 1637, and *Escherichia coli* KCTC 1923 grown at 37 °C—were grown overnight. They were then washed with phosphate-buffered saline (PBS) (10 mM, pH 7.5) and suspended in the same buffer to reach a final density of 1 × 10^6^ cfu ml^−1^. MIC values of protease SH21 against different microorganisms were represented in our previous report [8]. Protease SH21 at the appropriate MIC was introduced to the bacterial strains, and they were then incubated for 0, 2, 4, 6, 8, 10, and 12 h. The mixture was then filtered over a 0.22 um filter to remove the bacteria cells. After the filtrate had been suitably diluted, the optical density was measured at room temperature at 260 nm (Amersham Ultrospec 1100 Pro UV Vis, Hayward, CA, USA). To serve as control, bacterial cultures were incubated in PBS buffer (10 mM; pH 7.5).

### 3.9. PI Uptake Assay by Protease SH21

The protease SH21 membrane permeabilization experiment was carried out with propidium iodide (PI), a fluorescent dye [18]. In short, the bacteria *E. coli* ATCC 1923 and *S. aureus* KCTC 1928 were cultivated in MHB medium until they reached the mid-log phase, and then they were diluted to an OD600 value of 0.25 in sodium phosphate buffer (10 mM). Every bacteria was treated with PI (final concentration, 20 µM) in a dark 96-well plate. Each well was then mixed and then treated with protease SH21 at 1× MIC and 2× MIC. The percentage (%) of fluorescence intensity obtained from the protease SH21 was displayed in comparison with untreated controls. The fluorescence spectrophotometer was used to measure the absorbance at 580 nm and 620 nm for the excitation and emission, respectively.

### 3.10. Statistical Analysis

All tests were performed three times, and the results were presented as mean (±) standard deviation. The statistical analysis was evaluated using Student’s *t*-test or a one-way ANOVA.

## 4. Conclusions

In our previous study, we evaluated the antimicrobial activity of protease SH21 against both Gram-positive and Gram-negative strains. In our present work, we investigated the antimicrobial mode of action of protease SH21. In terms of mode of action, protease SH21 exhibited direct antimicrobial activity by disrupting the bacterial cell membrane. PI uptake assay also revealed that protease SH21 was able to integrate into the bacterial cell membrane. Based on the overall findings, we assume that protease SH21 may be used as a promising candidate for treating infectious illnesses caused by drug-resistant bacteria. Further study is needed to explore the diverse antimicrobial mechanism of Protease SH21.

## Figures and Tables

**Table 1:** Effect of pH and temperature on the antibacterial activity of protease SH21.

Treatment		Antimicrobial Activity (mm)
pH	3	11 ± 41
	6	14 ± 35
	9	16 ± 42
	12	0 ± 00
Temperature (°C)	30	16 ± 14
	40	16 ± 45
	50	16 ± 36
	60	15 ± 28
	70	14 ± 48
	80	13 ± 24
	100	12 ± 10
	121	0 ± 00

## Data Availability

Data are available on request from the author.

## References

[1] Ogunniran K.O. (2009). Antibacterial effects of extracts of Ocimum gratissimum and piper guineense on Escherichia coli and Staphylococcus aureus. Afr. J. Food Sci..

[2] Chopra I. (2007). Bacterial RNA polymerase: A promising target for the discovery of new antimicrobial agents. Curr. Opin. Investig. Drugs.

[3] Kuroda M., Ohta T., Uchiyama I., Baba T., Yuzawa H., Kobayashi I., Cui L., Oguchi A., Aoki K.-I., Nagai Y. (2001). Whole genome sequencing of meticillin-resistant Staphylococcus aureus. Lancet.

[4] Tersariol I.L.d.S., Pimenta D., Chagas J.R., Almeida P. (2002). Proteinase activity regulation by glycosaminoglycans. Braz. J. Med. Biol. Res..

[5] Secades P., Guijarro J. (1999). Purification and characterization of an extracellular protease from the fish pathogen Yersinia ruckeri and effect of culture conditions on production. Appl. Environ. Microbiol..

[6] Indarmawan T., Mustopa A.Z., Budiarto B.R., Tarman K. (2016). Antibacterial activity of extracellular protease isolated from an algicolous fungus Xylaria psidii KT30 against gram-positive bacteria. HAYATI J. Biosci..

[7] Rachanamol R., Lipton A., Thankamani V., Sarika A., Selvin J. (2017). Production of protease showing antibacterial activity by Bacillus subtilis VCDA associated with tropical marine sponge Callyspongia diffusa. J. Microb. Biochem. Technol..

[8] Tarek H., Nam K.B., Kim Y.K., Suchi S.A., Yoo J.C. (2023). Biochemical Characterization and Application of a Detergent Stable, Antimicrobial and Antibiofilm Potential Protease from Bacillus siamensis. Int. J. Mol. Sci..

[9] Tarek H., Cho S.S., Hossain M.S., Yoo J.C. (2023). Attenuation of oxidative damage via upregulating Nrf2/HO-1 signaling pathway by protease SH21 with exerting anti-inflammatory and anticancer properties in vitro. Cells.

[10] Creaser E. (1955). The induced (adaptive) biosynthesis of β-galactosidase in Staphylococcus aureus. Microbiology.

[11] Muthu S., Gopal V.B., Soundararajan S., Nattarayan K., Narayan K.S., Lakshmikanthan M., Malairaj S., Perumal P. (2017). Antibacterial serine protease from Wrightia tinctoria: Purification and characterization. Plant Physiol. Biochem..

[12] Benmebarek H., Escuder-Rodríguez J.-J., González-Siso M.-I., Karroub K. (2020). Test for the production and assay of the proteolytic activities of halophilic bacteria and archaea isolated from Algerian hypersaline environments. Proceedings.

[13] Marri L., Dallai R., Marchini D. (1996). The novel antibacterial peptide ceratotoxin A alters permeability of the inner and outer membrane of Escherichia coli K-12. Curr. Microbiol..

[14] Tsuji Y., Aoyama T., Takeuchi K., Homma K., Takahashi H., Nakajima Y., Shimada I., Natori S. (2001). Identification and characterization of an antibacterial peptide of the 26-kDa protease of Sarcophaga peregrina with antibacterial activity. J. Biochem..

[15] Wei C., Cui P., Liu X. (2023). Antibacterial Activity and Mechanism of Madecassic Acid against Staphylococcus aureus. Molecules.

[16] Miao J., Zhou J., Liu G., Chen F., Chen Y., Gao X., Dixon W., Song M., Xiao H., Cao Y. (2016). Membrane disruption and DNA binding of Staphylococcus aureus cell induced by a novel antimicrobial peptide produced by Lactobacillus paracasei subsp. tolerans FX-6. Food Control.

[17] Xiao J., Zhang H., Niu L., Wang X. (2011). Efficient screening of a novel antimicrobial peptide from Jatropha curcas by cell membrane affinity chromatography. J. Agric. Food Chem..

[18] Ko S.J., Kang N.H., Kim M.K., Park J., Park E., Park G.H., Kang T.W., Park J.B., Yi Y.E., Jeon S.H. (2019). Antibacterial and anti-biofilm activity, and mechanism of action of pleurocidin against drug resistant Staphylococcus aureus. Microb. Pathog..

